# Supplementary material for: Single-cell multiomics sequencing reveals the functional regulatory landscape of early embryos
Source: Nat Commun. 2021 Feb 23;12:1247. doi: 10.1038/s41467-021-21409-8 (PMC7902657; doi:10.1038/s41467-021-21409-8)
Supplement: Supplementary file 1 — Supplementary Information [file 41467_2021_21409_MOESM1_ESM.pdf]

Supplementary Information

a

	Zygote	2-cell	4-cell	L4-cell	8-cell	Morula	Blastocyst	total
RNA passed/ DNA failed	1	5	2	2	1	0	0	11
RNA failed/ DNA passed	0	0	0	0	0	0	8	8
Both failed	0	0	1	0	0	0	3	4
Both passed	12	19	45	22	31	24	57	210

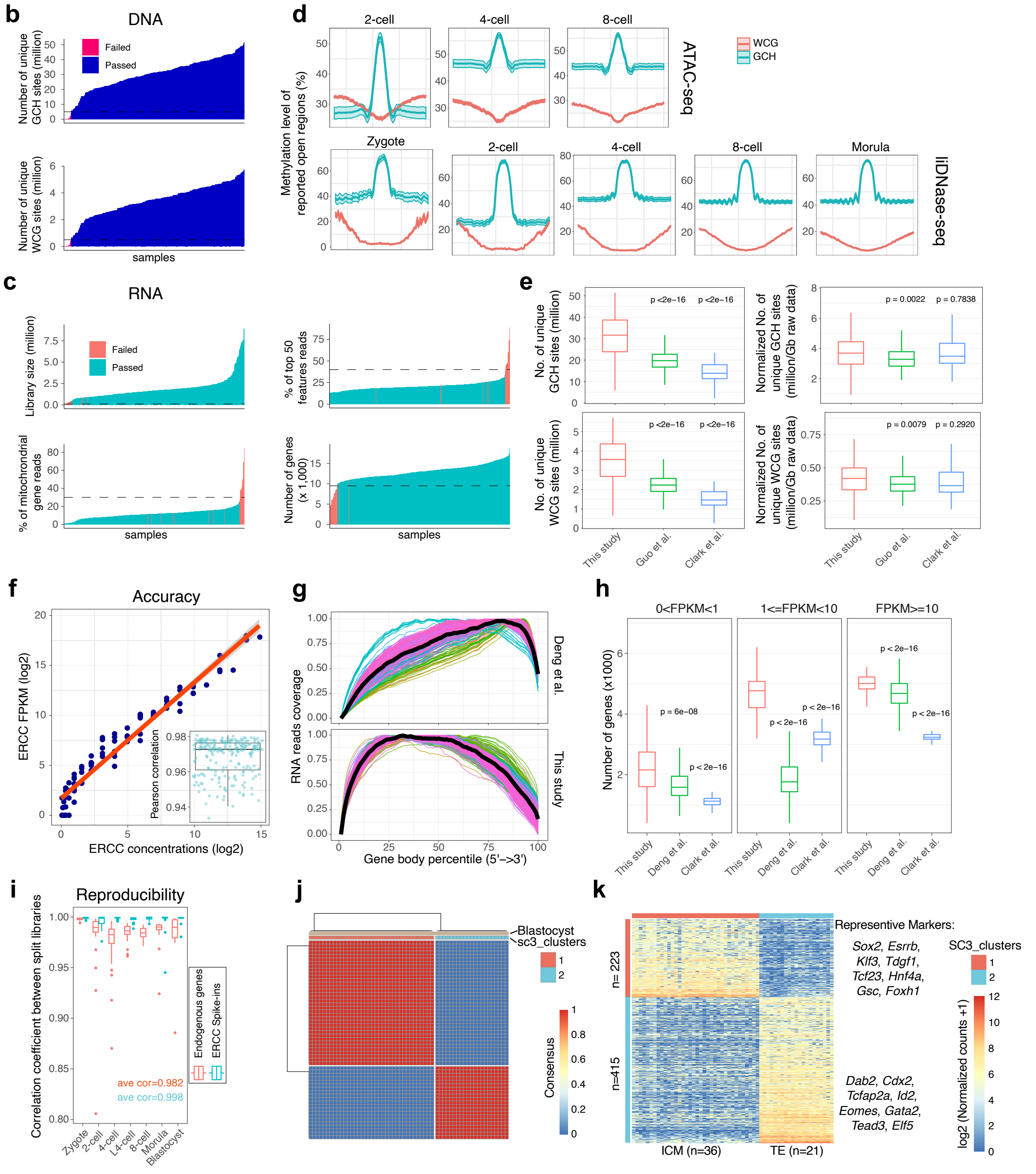

Supplementary Figure 1. Quality control of the scNOMeRe-seq datasets. a Summary of cell numbers in this study. Split RNA libraries from the same cell were merged, counting as one RNA library. **b-c** Quality control of DNA and RNA libraries. **d** DNA methylation level (WCG) and chromatin accessibility (GCH) around open regions defined by previously published studies (ref. 1-2). The error band showed 95%

confidence interval. **e** Box plot showing the number of unique GCH/WCG sites and sequencing depth normalized number of GCH/WCG sites in scCOOL-seq (ref. 3), scNMT-seq (ref. 4) and this study. *P* value, two-sided Student t-test. This study, n = 218 cells; Guo et al., n = 195 cells; Clark et al., n = 64 cells. **f** Scatter plot showing the relationship between expected ERCC spike-ins concentrations and detected expression level of a representative single cell. Box plot showing the Pearson correlation coefficients of cells passed quality control (n = 221 cells). **g** Profiles of RNA reads coverage along gene bodies in previously published data (ref. 5) and this study. Black lines showing the average rates. **h** Box plot showing the number of protein coding genes at different expression level of each single cell in previously published data (ref. 4-5) and this study. *P* value, two-sided Student t-test, compared with this study. This study, n = 221 cells; Deng et al., n = 264 cells; Clark et al., n = 74 cells. **i** Box plot showing the Pearson correlation coefficients of expression level (FPKM, log2) of endogenous genes or ERCC spike-ins between two split libraries of a single cell (n = 221 cells). **j** Consensus plot of E3.5 blastocyst (n = 57 cells) with gene expression level (log2 normalized counts). **k** Heat map showing gene expression level of ICM, TE lineage specific markers identified by SC3 for cluster 1 and 2, respectively. Representative markers are shown. Box plots showing median levels and the first and third quartile, whiskers indicate minimum and maximum values. Source data for **i** and **k** are provided as a Source Data file.

a

	Zygote	2-cell	4-cell	L4-cell	8-cell	Morula	ICM	TE	total
Normal	8/1	12/4	37/2	19/1	22	16	20	15	149/8
PG	4	3/1	4	3/1	7/1	0	0	0	21/3
Aneu	0	4	4	0	2	8	16	6	40
total	12/1	19/5	45/2	22/2	31/1	24	36	21	210/11

12 cells not included in this table: both QC failed, 4 cells; RNA QC failed cells at blastocyst stage, 8 cells.
Red colored number indicates only RNA data passed QC.

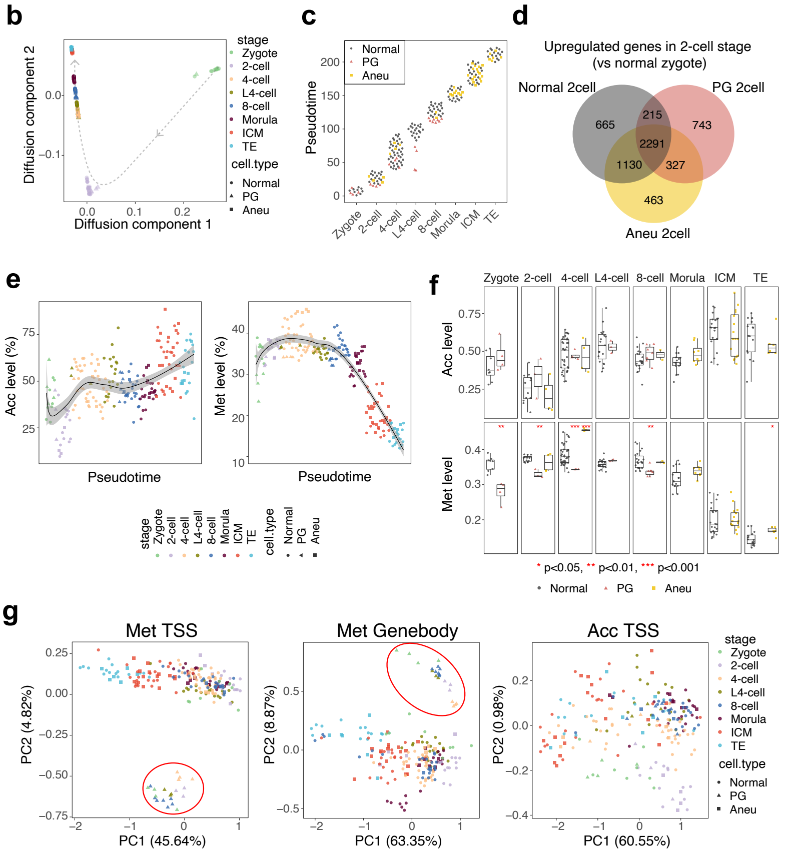

Supplementary Figure 2. Aberrant transcriptome and epigenomes of abnormal embryos. a Summary of numbers of Normal, PG and Aneu cells. PG, parthenogenetic; Aneu, aneuploid. **b** Developmental trajectory along preimplantation development inferred from the RNA data (n = 221 cells). **c** Dot plot showing cell orders along the pseudotime (y-axis) for each stage (x-axis). Total number of samples, n = 221 cells. **d** Venn diagram showing the overlaps of 2-cell embryos upregulated genes (2-cell blastomeres vs zygotes (n = 9 cells), FDR < 0.01, fold change > 2) among Normal (n = 16 cells), PG (n = 4 cells) and Aneu (n = 4 cells) blastomeres. **e** Dynamics of global chromatin accessibility (left) and DNA methylation level (right) of each individual cell (n = 210 cells) along developmental trajectory. The black lines show the regression fit. The error band showed 95% confidence interval. **f** Box plot showing the chromatin accessibility (top) and DNA methylation level (bottom) of Normal, PG and Aneu cells at each stage. Each box showing median levels and the first and third quartile, whiskers indicate minimum and maximum values. Two-sided Wilcox-test, Normal cells as control for each stage, * p < 0.05, ** p < 0.01, *** p < 0.001. **g** PCA analysis of DNA methylation level at TSS (left) and gene body (middle) regions as well as chromatin accessibility at TSS (right) regions for Normal, PG and Aneu blastomeres across preimplantation development. Red cycles

indicate the PG cells. The sample size of each group is provided in **a**. Source data for **f** are provided as a Source Data file.

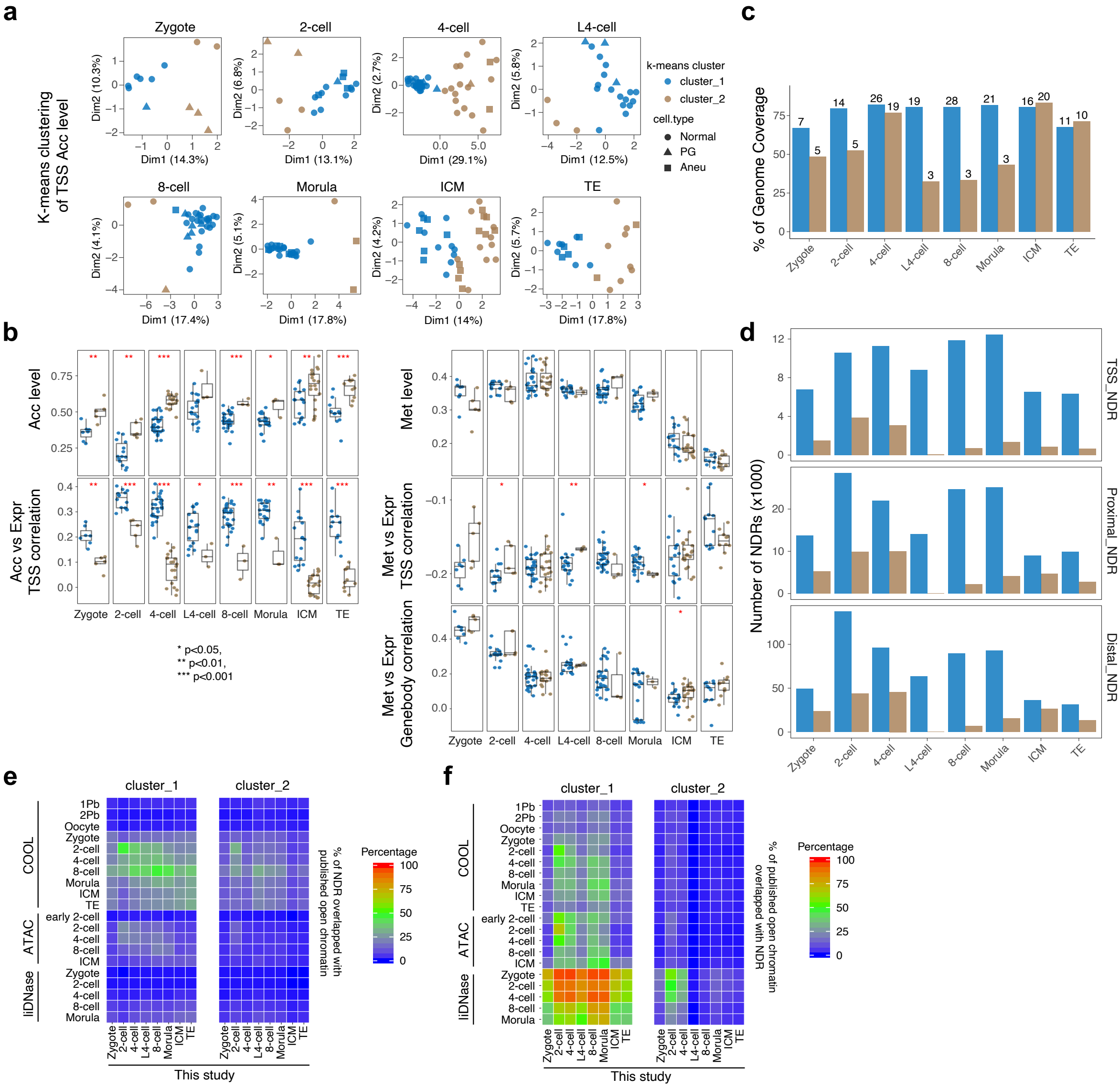

g

	Zygote	2-cell	4-cell	L4-cell	8-cell	Morula	ICM	TE	total
Expr	13	24	47	24	32	24	36	21	221
Met	8/4	16/3	41/4	19/3	24/7	24	36	21	189/21
Acc	7/5	14/5	26/19	19/3	28/3	21/3	16/20	11/10	142/68

The number of cells used / excluded for downstream analysis.

Supplementary Figure 3. scNOMeRe-seq uncovers different subgroups with specific chromatin accessibility pattern for each stage. a K-means clustering (k=2) of chromatin accessibility level at TSS regions for each stage. PG, parthenogenetic; Aneu, aneuploid. **b** Box plot showing the chromatin accessibility (left top), weighted Pearson correlation coefficients between chromatin accessibility at TSS regions and gene expression level (left bottom), DNA methylation level (right top), weighted Pearson correlation coefficients between DNA methylation level at TSS regions and gene expression level (right middle), weighted Pearson correlation coefficients between DNA methylation level at gene body regions and gene expression level (right bottom) for each k-means cluster at each stage. Each box showing median

levels and the first and third quartile, whiskers indicate minimum and maximum values. Two-sided Wilcox-test, * $p < 0.05$, ** $p < 0.01$, *** $p < 0.001$. **c** Bar plot showing the genomic coverage of unique GCH sites in each cluster at each stage. The number above each bar indicates the cell number of each cluster in each stage. **d** Bar plot showing the numbers of TSS_NDRs, Proximal_NDRs and Distal_NDRs in each cluster at each stage. **e** Heat map showing the percentages of NDRs overlapped with previously published open chromatin regions accounting for total NDRs detected in this study (ref. 1-3). **f** Heat map showing the percentages of previously published open chromatin regions overlapped with NDRs detected in this study accounting for total published open chromatin regions (ref. 1-3). **g** Summary of numbers of cells used (in black)/ excluded (in red) for downstream analysis. The sample size of each group is provided in **c**. Source data for **b** are provided as a Source Data file.

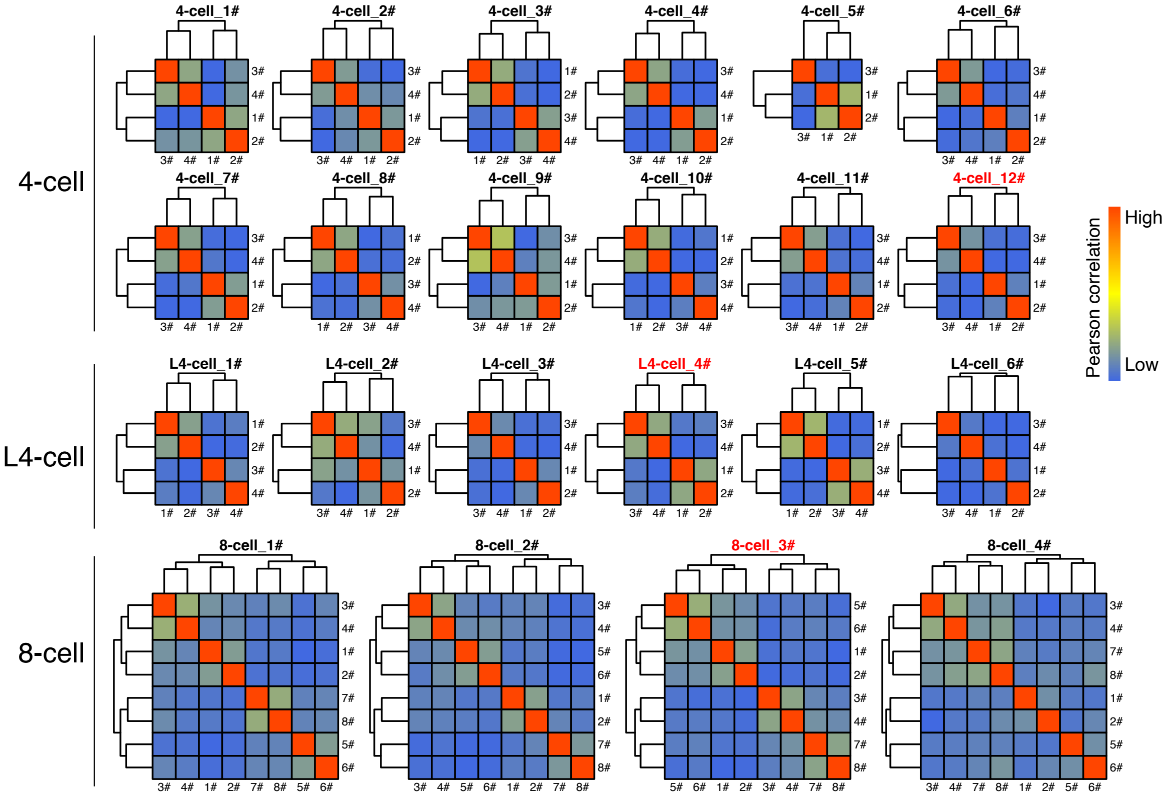

Supplementary Figure 4. Reconstruction of genetic lineages of early embryos using single-cell transcriptome datasets. Heat map showing the Pearson correlation coefficients of transcriptome level among cells in all embryos of 4-cell (n = 12 embryos), late 4-cell (n = 6 embryos) and 8-cell (n = 4 embryos) stages. Red labeled PG embryos.

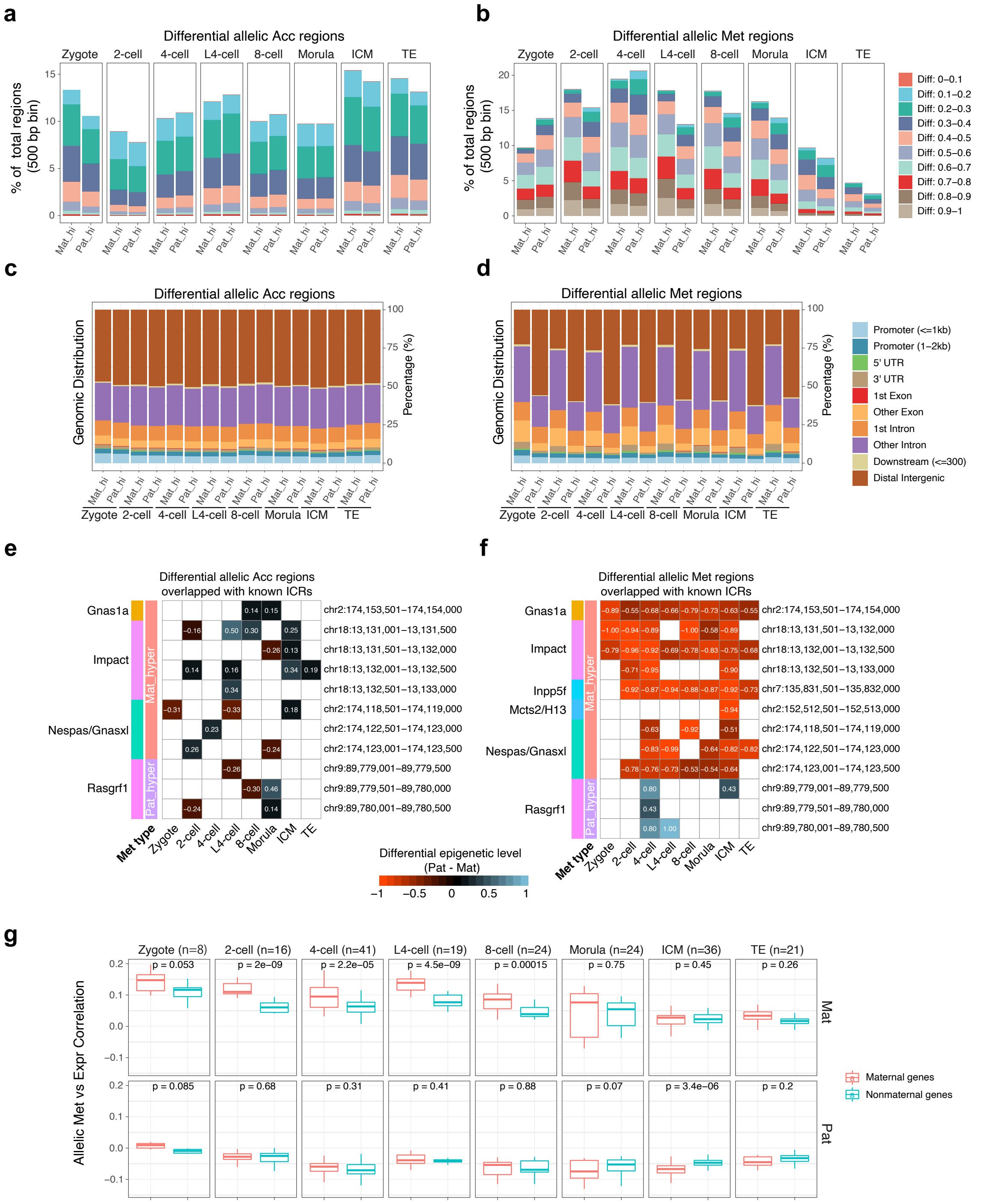

Supplementary Figure 5. Differential allelic epigenomes in early embryos. (a-b) Bar plot showing the fractions of differential (FDR < 0.01) allelic chromatin accessibility (**a**) and DNA methylation level (**b**) between maternal genome and paternal genome of each stage. Mat_hi, regions with higher maternal allelic level; Pat_hi, regions with higher paternal allelic level. (**c-d**) Bar plot showing the genomic distributions of differential allelic chromatin accessibility (**c**) and DNA methylation level (**d**) regions of each stage. (**e**) Heat map showing the difference of allelic chromatin accessibility at the differential allelic chromatin accessibility regions that overlap known imprinting control regions (ICRs). Pat_hyper, known paternal hypermethylated

ICRs; Mat_hyper, known maternal hypermethylated ICRs. The numbers indicate the differential chromatin accessibility level. (**f**) Heat map showing the difference of allelic DNA methylation level at the differential allelic DNA methylation regions that overlap known ICRs. Pat_hyper, known paternal hypermethylated ICRs; Mat_hyper, known maternal hypermethylated ICRs. The numbers indicate the differential DNA methylation level. (**g**) Box plot showing the Pearson correlations between DNA methylation level around genic regions of maternal allele and expression level of maternal genes vs nonmaternal genes. Each box showing median levels and the first and third quartile, whiskers indicate minimum and maximum values. P, p value, two-sided Student t-test. n, the cell numbers of each stage.

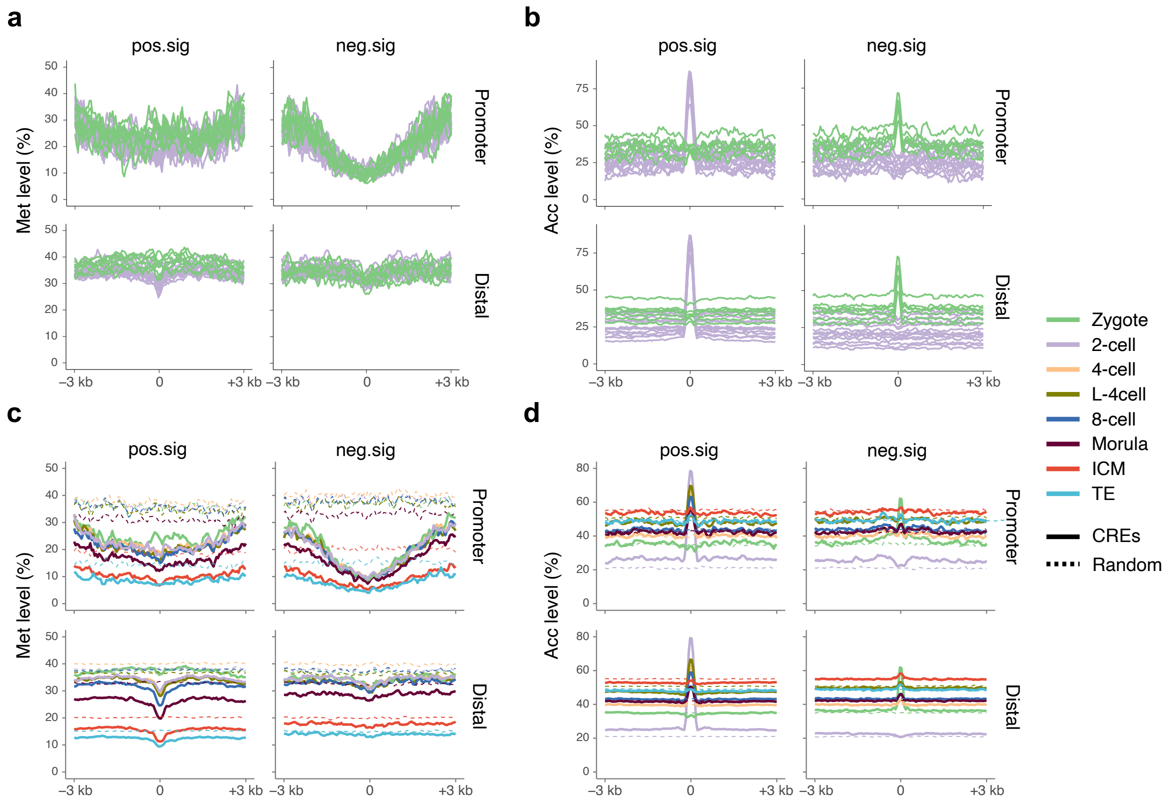

Supplementary Figure 6. Dynamics of DNA methylation and chromatin accessibility around ZGA associated CREs. a-b DNA methylation level (**a**) and chromatin accessibility (**b**) around ZGA associated CREs (from the upstream 3 kb of CRE center to the downstream 3 kb) for each individual cell of zygote and 2-cell embryos. For the Met data, Zygote, n = 8 cells; 2-cell, n = 16 cells. For the Acc data, Zygote, n = 7 cells; 2-cell, n = 14 cells. **c-d** DNA methylation level (**c**) and chromatin accessibility (**d**) around ZGA associated CREs and random regions for each preimplantation stage.

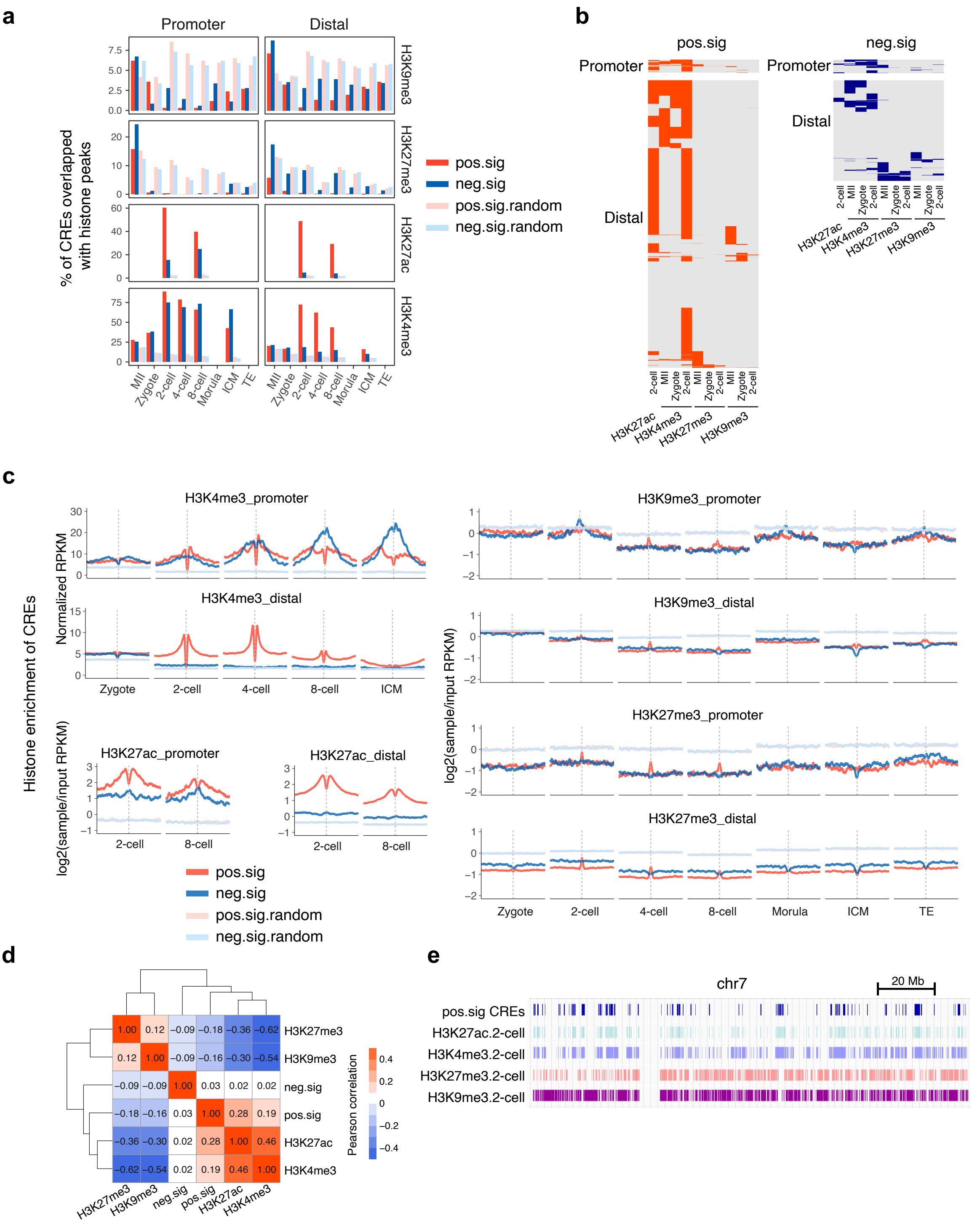

Supplementary Figure 7. Histone modification features of ZGA associated CREs. a Bar plot showing the percentage of histone modification peaks overlapped with ZGA associated CREs and random regions at promoter (left) and distal (right) regions for each stage. **b** Heat map showing the distribution of histone modification peaks at ZGA associated CREs. **c** Profiles showing the histone modification enrichment around ZGA associated CREs and random regions for each stage (from the upstream 3 kb of region center to the downstream 3 kb). **d** Heat map showing the colocalization among ZGA associated CREs and histone

modification peaks of H3K27ac, H3K4me3, H3K27me3 and H3K9me3 at 2-cell stage. The numbers showing the Pearson correlation coefficients. **e** IGV snapshot showing the distribution of ZGA positive-correlated CREs, histone modification peaks of H3K27ac, H3K4me3, H3K27me3 and H3K9me3 at 2-cell stage on chromosome 7.

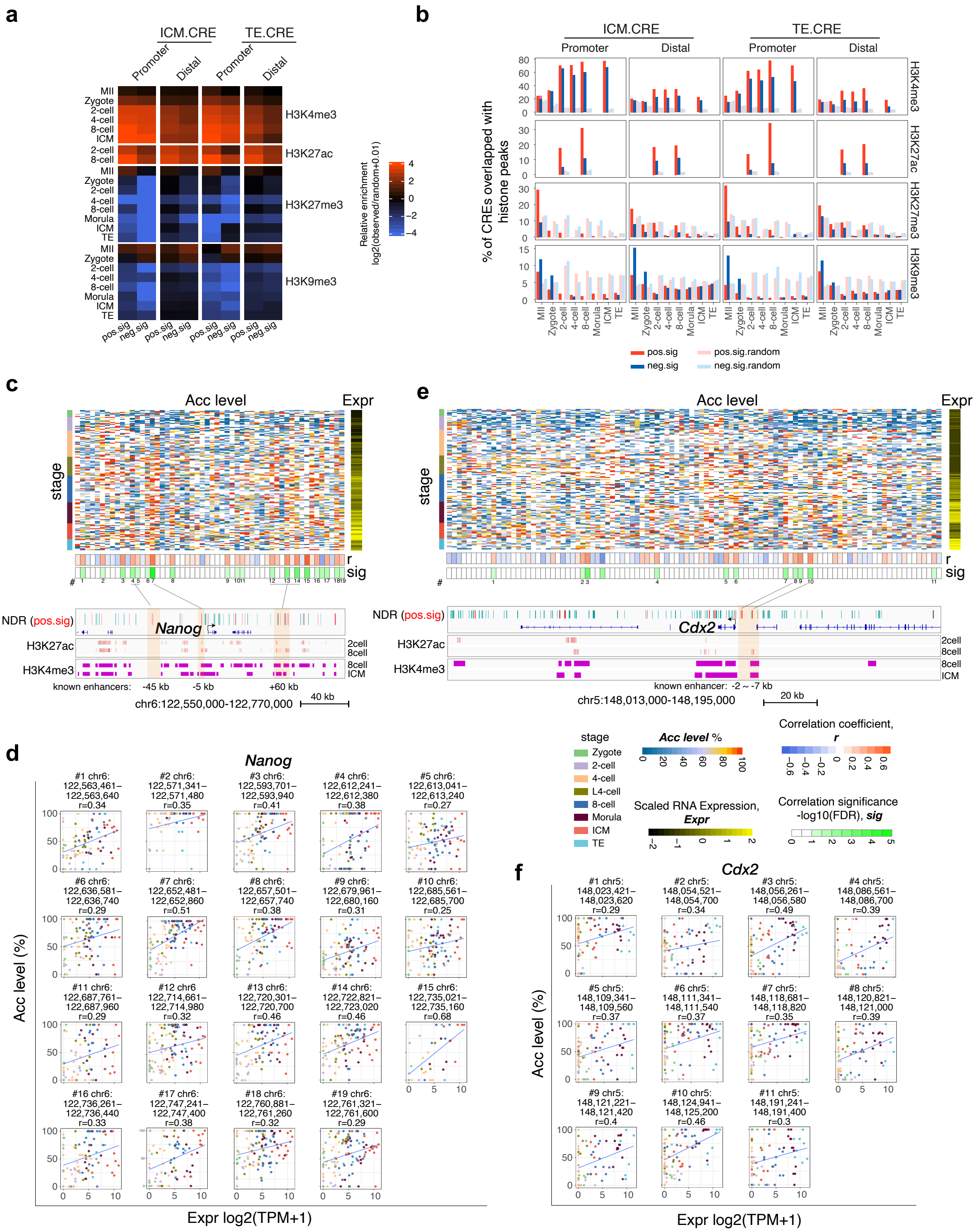

Supplementary Figure 8. Correlation analysis reveals ICM/TE associated CREs. a Heat map showing the enrichment of histone modifications in ICM.CREs (left) and TE.CREs (right). The enrichment was calculated as log2 ratio for the number of observed CREs that overlap with histone modification peaks divided by the number of random regions that overlap with histone modification peaks. **b** Bar plot showing the percentage of ICM.CREs (left) and TE.CREs (right) overlapped with histone modification peaks for each stage. Random regions are also shown. (**c** and **e**) (top) Heat map showing chromatin accessibility (Acc level)

of *Nanog* (**c**) or *Cdx2* (**e**) gene locus surrounding NDRs (from TSS upstream 100 kb to TES downstream 100 kb), and expression level (Expr, scaled log2(TPM+1)) of *Nanog* (**c**) or *Cdx2* (**e**) in each individual cell of early embryos; the weighted Pearson correlation coefficients (r) and significances (sig) are shown. #, the labels of positive-correlated CREs. (bottom) IGV snapshot showing the distribution of *Nanog* (**c**) or *Cdx2* (**e**) surrounding NDRs (positive-correlated CREs are in red), H3K27ac and H3K4me3 peaks. Known enhancers of *Nanog* (**c**) or *Cdx2* (**e**) are shaded. (**d** and **f**) Scatter plot showing the expression level of *Nanog* (**d**) or *Cdx2* (**f**) and chromatin accessibility of positive-correlated CREs which have been labeled in (**c**) for *Nanog* and (**e**) for *Cdx2*. The genomic coordinates of CREs and the correlation coefficients are shown. Sample size of each group for **c-f** is proved in Supplementary Fig. 3**g**.

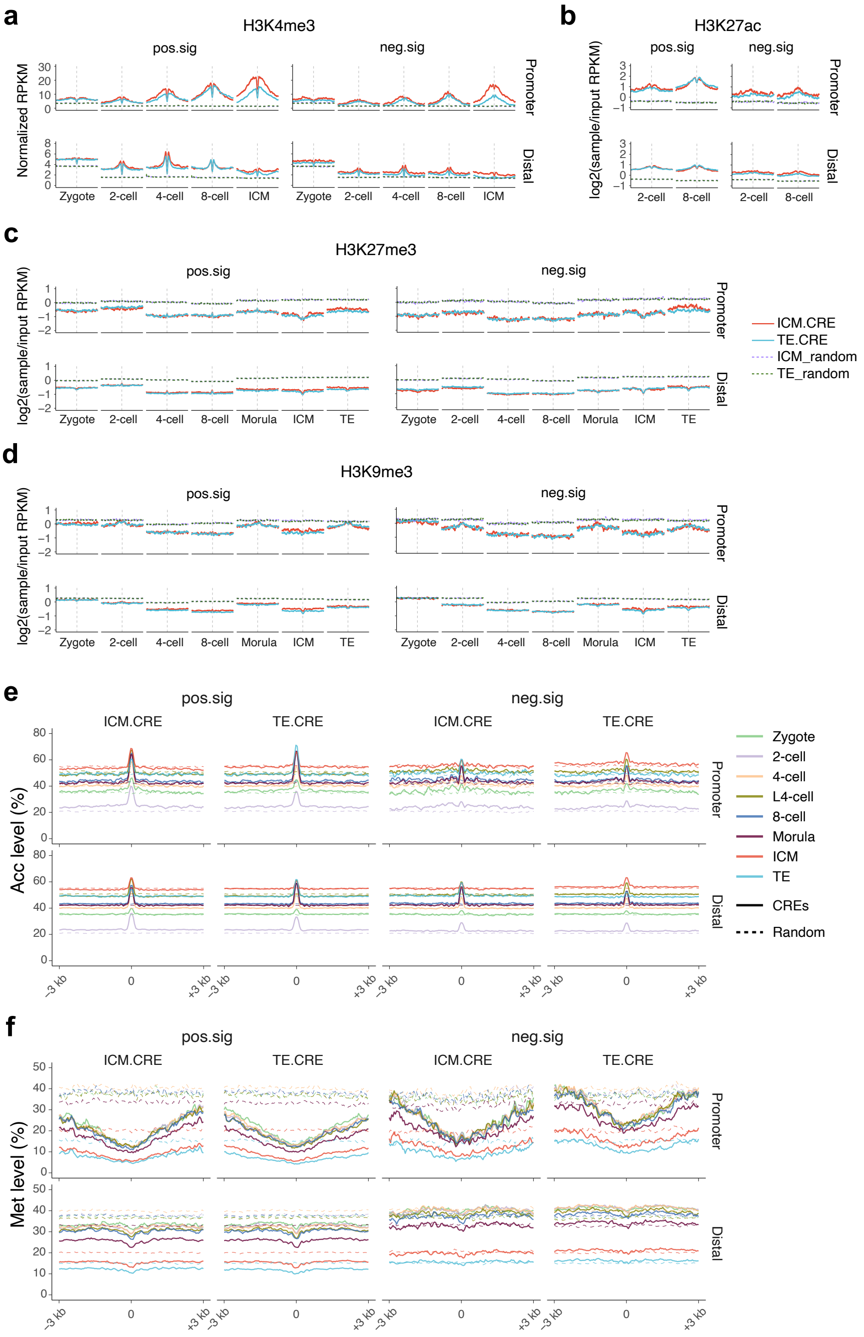

Supplementary Figure 9. Dynamics of epigenetic level around ICM/TE associated CREs. a-d Profiles showing the histone modification enrichment around ICM/TE associated CREs and random regions for each stage (from the upstream 3 kb of region center to the downstream 3 kb). **e-f** Chromatin accessibility (**e**) and DNA methylation level (**f**) around ICM/TE associated CREs and random regions for each stage.

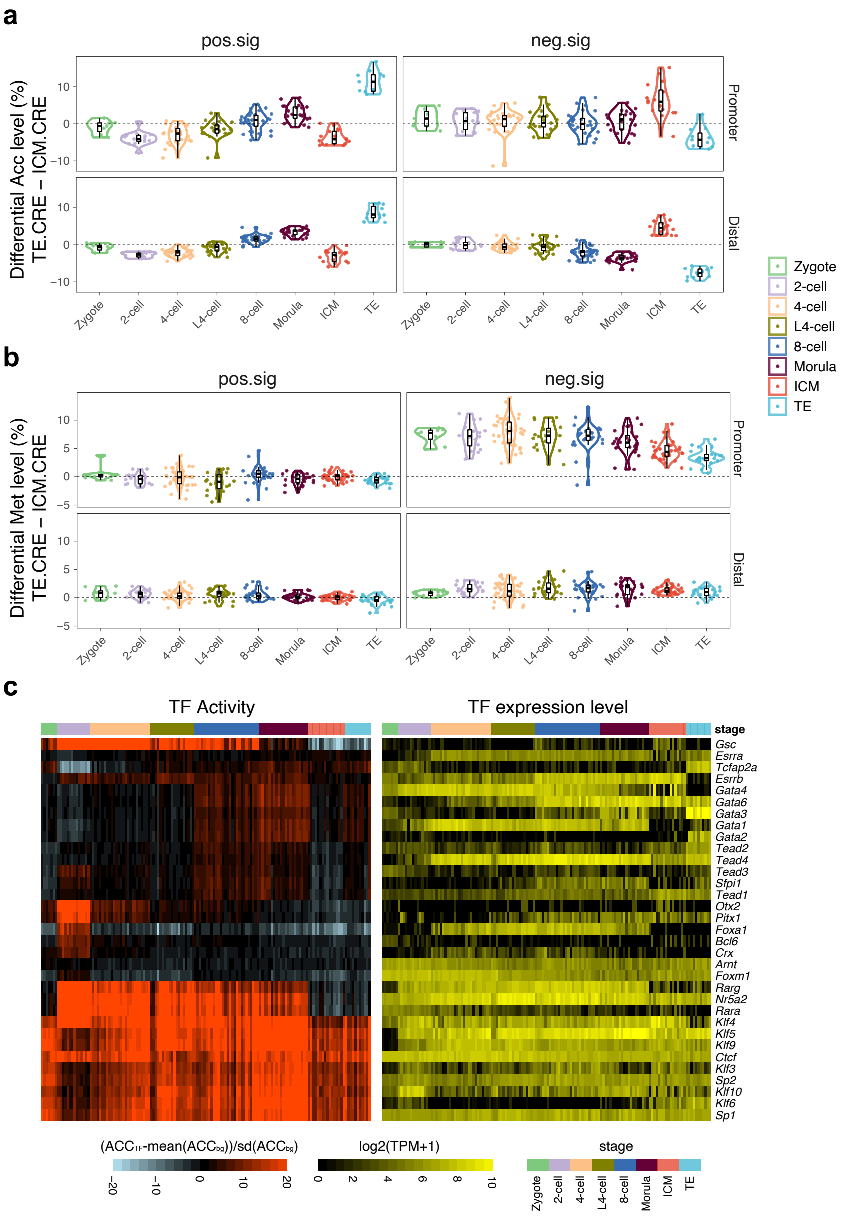

Supplementary Figure 10. Unsynchronized activation of ICM/TE associated CREs during preimplantation development. a-b Violin plot showing the global difference of chromatin accessibility (**a**) and DNA methylation level (**b**) between ICM.CRE and TE.CRE in each individual cell of each stage. Each box showing median levels and the first and third quartile, whiskers indicate minimum and maximum values. **c** Heat map showing the TF activity (left) and RNA expression level (right) of ICM.CREs and TE.CREs enriched TFs in each individual cell across preimplantation development. Sample size of each group is proved in Supplementary Fig. 3**g**. Source data for **a-c** are provided as a Source Data file.

Supplementary References

1 Wu, J. *et al.* The landscape of accessible chromatin in mammalian preimplantation embryos. *Nature* **534**, 652-657, doi:10.1038/nature18606 (2016).

2 Lu, F. *et al.* Establishing Chromatin Regulatory Landscape during Mouse Preimplantation Development. *Cell* **165**, 1375-1388, doi:10.1016/j.cell.2016.05.050 (2016).

3 Guo, F. *et al.* Single-cell multi-omics sequencing of mouse early embryos and embryonic stem cells. *Cell Res* **27**, 967-988, doi:10.1038/cr.2017.82 (2017).

4 Clark, S. J. *et al.* scNMT-seq enables joint profiling of chromatin accessibility DNA methylation and transcription in single cells. *Nat Commun* **9**, 781, doi:10.1038/s41467-018-03149-4 (2018).

5 Deng, Q., Ramskold, D., Reinius, B. & Sandberg, R. Single-cell RNA-seq reveals dynamic, random monoallelic gene expression in mammalian cells. *Science* **343**, 193-196, doi:10.1126/science.1245316 (2014).
