# Supplementary material for: Single-cell multiomics sequencing reveals the functional regulatory landscape of early embryos
Source: Nat Commun. 2021 Feb 23;12:1247. doi: 10.1038/s41467-021-21409-8 (PMC7902657; doi:10.1038/s41467-021-21409-8)
Supplement: Supplementary file 3 — Description of Additional Supplementary Files [file 41467_2021_21409_MOESM3_ESM.pdf]

Description of Additional Supplementary Files

Supplementary Data 1: scNOMeRe-seq Primer Sequences.

Supplementary Data 2: Sample and Sequencing Information. Sheet 1. scNOMeRe-seq sample information; Sheet 2-4. RNA-seq, NOMe-seq and BS-seq data sequencing information; Sheet 5-6. NOMe-seq and BS-seq covered WCG/GCH sites information.

Supplementary Data 3: ZGA Genes. List of 2-cell embryos significantly upregulated genes, compared to zygotes. The Wald test was used for significance testing in DESeq2. The P values were adjusted for multiple testing using the procedure of Benjamini-Hochberg.

Supplementary Data 4: ZGA associated CREs. Sheet 1-2. ZGA genes associated promoter and distal CREs. The significance of the coefficients was calculated with two-tailed Student's t-tests. The P values were further adjusted by the BenjaminiHochberg approach.

Supplementary Data 5: DEGs between ICM and TE. The Wald test was used for significance testing in DESeq2. The P values were adjusted for multiple testing using the procedure of BenjaminiHochberg.

Supplementary Data 6: ICM and TE associated CREs. Sheet 1-2. ICM/TE-DEGs associated promoter and distal CREs. The significance of the coefficients was calculated with two-tailed Student's t-tests. The P values were further adjusted by the BenjaminiHochberg approach.
