# Supplementary material for: Single-cell multiomics sequencing reveals the functional regulatory landscape of early embryos
Source: Nat Commun. 2021 Feb 23;12:1247. doi: 10.1038/s41467-021-21409-8 (PMC7902657; doi:10.1038/s41467-021-21409-8)
Supplement: Supplementary file 10 — Reporting Summary [file 41467_2021_21409_MOESM10_ESM.pdf]

nature research

Corresponding author(s): Jie Qiao, Liying Yan

Last updated by author(s): Dec 4, 2020

Reporting Summary

Nature Research wishes to improve the reproducibility of the work that we publish. This form provides structure for consistency and transparency in reporting. For further information on Nature Research policies, see our Editorial Policies and the Editorial Policy Checklist.

Statistics

For all statistical analyses, confirm that the following items are present in the figure legend, table legend, main text, or Methods section.

n/a	Confirmed	
[ ]	[x]	The exact sample size (*n*) for each experimental group/condition, given as a discrete number and unit of measurement
[ ]	[x]	A statement on whether measurements were taken from distinct samples or whether the same sample was measured repeatedly
[ ]	[x]	The statistical test(s) used AND whether they are one- or two-sided<br>*Only common tests should be described solely by name; describe more complex techniques in the Methods section.*
[x]	[ ]	A description of all covariates tested
[ ]	[x]	A description of any assumptions or corrections, such as tests of normality and adjustment for multiple comparisons
[ ]	[x]	A full description of the statistical parameters including central tendency (e.g. means) or other basic estimates (e.g. regression coefficient) AND variation (e.g. standard deviation) or associated estimates of uncertainty (e.g. confidence intervals)
[ ]	[x]	For null hypothesis testing, the test statistic (e.g. *F*, *t*, *r*) with confidence intervals, effect sizes, degrees of freedom and *P* value noted<br>*Give P values as exact values whenever suitable.*
[x]	[ ]	For Bayesian analysis, information on the choice of priors and Markov chain Monte Carlo settings
[x]	[ ]	For hierarchical and complex designs, identification of the appropriate level for tests and full reporting of outcomes
[ ]	[x]	Estimates of effect sizes (e.g. Cohen's *d*, Pearson's *r*), indicating how they were calculated

Our web collection on statistics for biologists contains articles on many of the points above.

Software and code

Policy information about availability of computer code

Data collection	Sequencing was performed using an Illumina HiSeq X Ten platform with 150 bp paired-end reads.
Data analysis	For RNA data, Trim Galore (v0.4.4) and STAR (v2.5.3a) were used to do the quality control and alignment. After aligment, RSeQC (v2.6.4) were used to remove the rRNA reads and featureCounts (v1.5.3) was used to obtain the counts of each gene. The differentially expressed genes were identified by DESeq2 (v1.24.0). PCA was performed using pcaMethods (v1.76.0) and the hclust (within R v3.6.0) function with the ward.D2 method was used for unsupervised hierarchical clustering in R package. SC3 (v1.12.0) package in R was used to determine the cell cluster. Destiny (v2.14.0) package in R was used to construct the psudotime.<br>For DNA data, Trim Galore (v0.4.4) and Bismark (v0.18.2, with bowtie2 v2.3.2) were used to do the quality control and alignment. R package HMMcopy (v1.24.0) was used to deduce CNV in DNA data. findMotifsGenome.pl in HOMER (v4.10.3) was used to identify enriched TF motifs in CREs with parameters "-size given -len 8,10,12".<br>Custom codes were written in Python, Perl and R and were available upon request.

For manuscripts utilizing custom algorithms or software that are central to the research but not yet described in published literature, software must be made available to editors and reviewers. We strongly encourage code deposition in a community repository (e.g. GitHub). See the Nature Research guidelines for submitting code & software for further information.

Data

Policy information about availability of data

All manuscripts must include a data availability statement. This statement should provide the following information, where applicable:

- Accession codes, unique identifiers, or web links for publicly available datasets
- A list of figures that have associated raw data
- A description of any restrictions on data availability

Raw sequencing data and processed files (RNA raw counts, WCG and GCH bigwig files) in this study have been deposited in the Gene Expression Omnibus (GEO) with the accession number GSE136718. Publicly available datasets were downloaded from the GEO database (GSE71434 for H3K4me3 modifications; GSE97778 for H3K9me3 modifications; GSE72784 for H3K27ac modifications; GSE73952 for H3K27me3 modifications). Source data for figures 2h, 4e, 5g and supplementary figures 1i, 1k, 2f, 3b, and 10a-c are provided with this paper.

Field-specific reporting

Please select the one below that is the best fit for your research. If you are not sure, read the appropriate sections before making your selection.

- [x] Life sciences
- [ ] Behavioural & social sciences
- [ ] Ecological, evolutionary & environmental sciences

For a reference copy of the document with all sections, see nature.com/documents/nr-reporting-summary-flat.pdf

Life sciences study design

All studies must disclose on these points even when the disclosure is negative.

Sample size	In total, 229 cells were retained after quality control, containing 221 single cell RNA data and 218 single cell DNA data. All the stages in our study had more than 12 cells with both RNA data and DNA data, as shown in Extended Data Figure 1a. According to previously published paper (Guo et al., 2017, Cell Research), 4 cells was sufficient to evaluate the openness. Therefore, the sample size in this study is sufficient to support our findings.
Data exclusions	For sequencing data, the low-quality cells were excluded according to the criteria described in Methods. The exclusion criteria were pre-established.
Replication	For each stage, at least three embryos were collected. The number of biological replicates in each stage was shown in Extended Data Figure 1a, and more detailed information of all replicates can be found in Supplementary Table 1. The results were consistent across replicates of each embryo stage.
Randomization	This is not applicable for this study. All single cells were allocated into different groups according to their development stages.
Blinding	Not applicable for this study since no specific grouping. The single cells were collected at different time points as the researchers need to be known to the relatively precise development stage while handling the embryos to reduce the batch effect.

Reporting for specific materials, systems and methods

We require information from authors about some types of materials, experimental systems and methods used in many studies. Here, indicate whether each material, system or method listed is relevant to your study. If you are not sure if a list item applies to your research, read the appropriate section before selecting a response.

Materials & experimental systems

n/a	Involved in the study	
[x]	[ ]	Antibodies
[x]	[ ]	Eukaryotic cell lines
[x]	[ ]	Palaeontology and archaeology
[ ]	[x]	Animals and other organisms
[x]	[ ]	Human research participants
[x]	[ ]	Clinical data
[x]	[ ]	Dual use research of concern

Methods

n/a	Involved in the study	
[x]	[ ]	ChIP-seq
[x]	[ ]	Flow cytometry
[x]	[ ]	MRI-based neuroimaging

Animals and other organisms

Policy information about studies involving animals; ARRIVE guidelines recommended for reporting animal research

Laboratory animals	The female mice used in this study were 6- to 8-week-old B6D2F1/J (BDF1) mice, and the male mice were 12-week-old 129S1 mice. All the mice used in study were were purchased from and housed in Department of Experimental Animal Sciences, Peking University

	Health Science Center, Beijing, China.
Wild animals	This study didn't involve wild animals.
Field-collected samples	This study didn't involve field-collected samples.
Ethics oversight	All animal-related experimental procedures were carried out under ethical guidelines set forth by the Animal Care and Use Committee of Peking University Health Science Center.

Note that full information on the approval of the study protocol must also be provided in the manuscript.
